# Biomechanical Effects of the Badminton Split-Step on Forecourt Lunging Footwork

**DOI:** 10.3390/bioengineering11050501

**Published:** 2024-05-17

**Authors:** Yile Wang, Liu Xu, Hanhui Jiang, Lin Yu, Hanzhang Wu, Qichang Mei

**Affiliations:** 1 Faculty of Sports Science, Ningbo University, Ningbo 315211, China; 2 Auckland Bioengineering Institute, University of Auckland, Auckland 1010, New Zealand

**Keywords:** badminton, split-step, lunge, biomechanics, lower limb

## Abstract

Background: This research investigates the biomechanical impact of the split-step technique on forehand and backhand lunges in badminton, aiming to enhance players’ on-court movement efficiency. Despite the importance of agile positioning in badminton, the specific contributions of the split-step to the biomechanical impact of lunging footwork still need to be determined. Methods: This study examined the lower limb kinematics and ground reaction forces of 18 male badminton players performing forehand and backhand lunges. Data were collected using the VICON motion capture system and Kistler force platforms. Variability in biomechanical characteristics was assessed using paired-sample *t*-tests and Statistical Parametric Mapping 1D (SPM1D). Results: The study demonstrates that the split-step technique in badminton lunges significantly affects lower limb biomechanics. During forehand lunges, the split-step increases hip abduction and rotation while decreasing knee flexion at foot contact. In backhand lunges, it increases knee rotation and decreases ankle rotation. Additionally, the split-step enhances the loading rate of the initial ground reaction force peak and narrows the time gap between the first two peaks. Conclusions: These findings underscore the split-step’s potential in optimizing lunging techniques, improving performance and reducing injury risks in badminton athletes.

## 1. Introduction

Badminton, as a widely popular sport globally, attracts numerous enthusiasts and professional athletes due to its fast-paced, agile, and highly skilled nature [1,2,3]. In badminton matches, athletes are required to swiftly react to the opponents’ shots and quickly maneuver to appropriate positions for a counterattack. Efficient badminton footwork techniques, such as jump landing, split-step, forehand and backhand lunging steps, cross steps, lateral shuffles, rapid net shots, and turning, play a crucial role in athletes’ movement efficiency and shot quality [1,4,5]. In this process, athletes must rapidly initiate and adeptly employ a series of complex footwork combinations, such as initiating with small steps followed by cross steps, adjustment steps, large strides, propulsion steps, jump steps, and take-off steps, to swiftly react to the incoming shuttlecock. This technique, known as the split-step, involves utilizing leg strength to pre-step in the initial phase of executing movement footwork to enhance the quality of movement footwork [6].

In badminton singles, players frequently lunge forward to hit the shuttlecock, accounting for approximately 37% of all movements [1,7,8,9], which require athletes to possess rapid mobility, as well as excellent coordination and strength control, to ensure stability upon reaching the striking position for accurate shot execution. Athletes typically employ the split-step during forecourt lunges to attain better initial velocity and advantageous positioning. The split-step is a crucial preparatory action, aiding athletes in swiftly transitioning from a stationary to a dynamic state, providing impetus and direction for subsequent lunging movements.

The biomechanical characteristics of lunging steps and their impact on athletes’ performance have been widely discussed. Yu et al. (2021) further investigated the effects of different lunge step directions (such as left forward, right forward, left backward, and right backward) on patellofemoral joint load, revealing that left backward lunging exhibited higher contact pressure and von Mises stress, particularly on the patellar cartilage. These studies provide crucial insights into understanding the biomechanical properties of lunging steps [10]. Mei et al. (2017) explored the biomechanical characteristics of badminton players with different skill levels during right-forward lunging, finding significant differences in knee joint moments and ground reaction forces between professional and amateur players [11]. Additionally, Lam et al. (2017) indicated that heel design influences ground reaction forces and knee joint moments during maximum lunge steps for elite and intermediate badminton players, suggesting that athletes’ skill levels and footwear design may affect the biomechanical characteristics of lunging steps [12]. Kuntze et al. (2010) examined the mechanical attributes of top male badminton players during specific movement techniques such as lunging, stepping, and shuffling through video analysis and biomechanical methods [13].

The split-step technique is common in racket sports such as tennis and badminton. Aviles et al. (2002) found in their study that high-level tennis players always execute a split-step (preparatory movement) before serving or receiving serves [6]. According to Phomsoupha et al. (2018), the split-step enables athletes to effectively utilize elastic energy in subsequent movements through the stretch-shortening cycle (SSC) mechanism of muscles [14]. Furthermore, Filipčič et al. (2017) conducted a comparative analysis of professional and junior badminton players and observed that professional players demonstrate more significant pre-activation of lower limb muscles during the execution of the split-step, facilitating faster initiation and higher acceleration in subsequent movements [15]. Uzu et al. (2009) analyzed the timing and frequency of split-steps in badminton matches and found that executing the split-step immediately after the opponent’s shot is most effective, aiding athletes in adjusting to optimal positions in the shortest time possible [16]. Hsueh et al. (2016) pointed out that due to immature physical development and neuromuscular control, the efficiency of split-step execution in adolescent athletes is generally lower compared to adult professional athletes [17]. Regarding gender differences, Mecheri et al. (2019) discovered in their study that male athletes outperform females in generating power during the split-step, while females exhibit better flexibility in footwork [18].

Despite the valuable insights provided by previous research on badminton footwork techniques, the significance of the split-step as the initiating phase of footwork execution is undeniable. However, there remains limited research on the specific influence of the split-step on the biomechanical characteristics of lunging steps. This necessitates a deeper understanding of the mechanism behind the split-step technique. Therefore, this study aims to conduct detailed measurements and analysis of badminton players’ kinematic parameters and ground reaction forces during lunging steps with and without the split-step technique through experimental methods. The objective is to elucidate the impact of the split-step technique on the lower limb biomechanical characteristics of athletes.

This study aims to investigate the biomechanical characteristics of the lower limbs of badminton players during forehand and backhand lunging steps with and without the split-step technique. By measuring and analyzing parameters such as kinematics and ground reaction forces during lunging steps in both scenarios, this research seeks to elucidate the mechanism of the split-step in badminton and how it affects athletes’ movement efficiency and stability. Additionally, this study will explore the potential value of the split-step technique in preventing sports injuries, providing coaches and athletes with more scientific and rational training guidance.

## 2. Materials and Methods

### 2.1. Participants

The sample size was calculated using GPower v3.1 [19] with an ANOVA F test for repeated measures within factors of a lateral wedge with incremental hardness, with an effect size (f) of 0.5, a level of 0.05, and a power value of 0.996. This study recruited a total of 18 male participants who were university-level badminton players (age: 24.51 ± 1.30 years, mass: 66.47 ± 8.42 kg, height: 172.60 ± 7.65 cm, BMI: 22.31 ± 3.21 kg/m^2^, years of playing: 7.07 ± 2.89 years). All participants were right-handed. They were required to meet the following criteria: (1) have a minimum of three years of experience in playing badminton, engaging in badminton training or competitive activities at least 2–3 times per week; (2) have no lower limb or whole-body deformities; and (3) have been free from injury or illness for the past six months prior to the start of the experiment, with no lower limb injuries. Participants provided informed consent before the experiment, demonstrating their understanding of the experimental procedures and objectives. Pre-experimental trials were conducted according to the experimental protocol.

Participants refrained from undertaking high-intensity training or competitive activities for two days preceding the experiment. To mitigate the potential confounding influence of footwear, each participant was provided with identical badminton shoes of the same brand and type [11,20].

The study was approved by the ethics committee of the research institute at the university, and all participants were informed of the test objectives, procedures, and requirements with written consent.

### 2.2. Experimental Protocol

**Figure 1:**
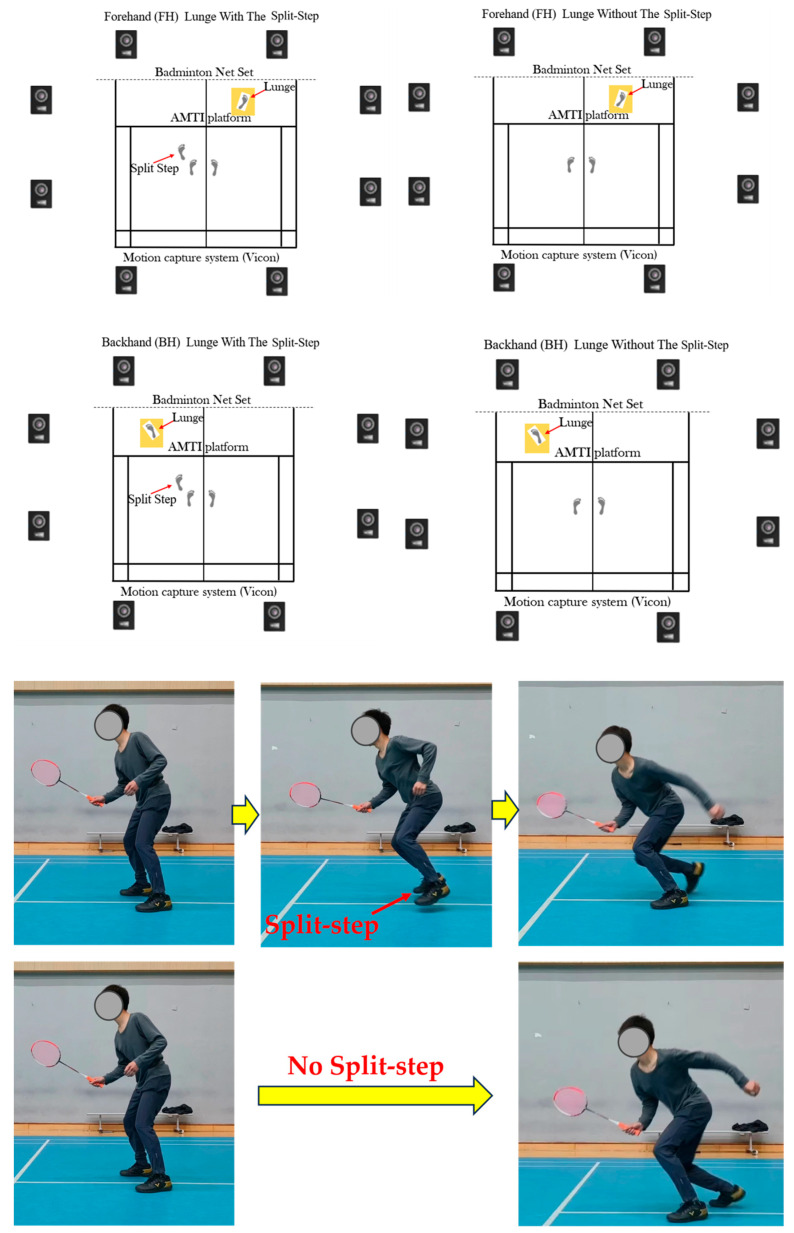
Illustration of experimental setup and the (non) split-step lunging footwork.

Forward forehand (FH) and backhand lunges (BH) are two of the most critical forward lunge techniques [9,20,21]. Following previous research, the forehand lunge is characterized by moving in the direction of the racket hand, orienting the chest towards the net, executing a stroke with the racket, and promptly returning to the initial position [20]. Each lunge should ideally be accomplished within a 3 s timeframe, covering a distance approximately 1.5 times the length of the leg. On the other hand, the backhand lunge entails having the back oriented towards the net [11,12,20]. More specific details of the two footwork and lab setup are illustrated in Figure 1.

All participants were experienced players with right-sided dominance for racquet grasp and right leg performing lunges, as badminton footwork typically involves unilateral hand and foot [3,5,10]. Specifically, all badminton players initiated the FH and BH lunges with a split-step, stepping up the left foot, followed by the right leg and foot for lunges to the proper forecourt or the left forecourt. Then, all badminton players initiated an FH and BH lunge without making a split-step, followed by a right leg and foot lunge to either the right or left forecourt. Thus, the lower limb of interest for lunging footwork was the right side.

After determining the experimental test action, a lab-simulated badminton court facilitated with an 8-camera Vicon motion capture system and synchronously connected AMTI force plates was set up for the biomechanical experiment to record the markers’ positions and ground reaction forces during badminton footwork [5,10,11]. The data collection frequencies were 200 Hz and 1000 Hz, respectively. The marker set model in this study included markers to both acromia of the torso, bilateral ASIS and PSIS of the pelvis, 3-marker cluster to the lateral aspect of both thighs, medial and lateral knee epicondyles, 3-marker cluster to the lateral aspect of both shank, medial, and lateral ankle malleoli, posterior calcaneus, anterior toe-tip, medial M1, and lateral M5 of the bilateral lower limb. The model was employed and validated in our previous study of badminton directional lunges [5,10].

The lab setup included a badminton net and stick-hang shuttlecock in the target region for lunges to mimic real court situations. Before the data collection, badminton players were required to perform warm-up and lab court familiarization practice with randomly selected footwear for 10 min. Lunges were performed to standard and visually supervised by an experienced coach. Approach speed was defined as the speed from the initial position to force plate foot contact [22], which was manually controlled with a stopwatch by the coach.

### 2.3. Data Processing

**Figure 2:**
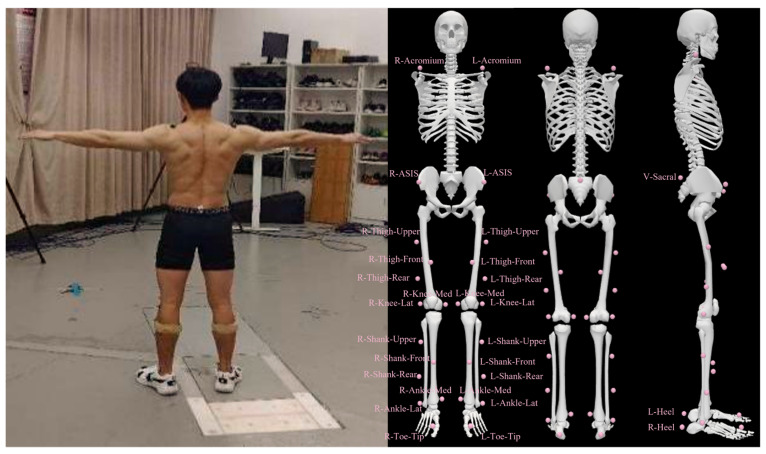
Diagram of marker set placement.

The target limb for FH and BH footwork was the right limb. This study aimed to investigate the effect of the split-step on the performance of the badminton net lunge. Thus, as a close chain, the stance phase focused on analyzing the contact times, joint angles, and vertical ground reaction force (VGRF), defined from the threshold of 20 N in vertical ground reaction force [5,10]. Given their velocity sensitivity, the velocity was regulated utilizing a stopwatch to account for its impact on biomechanical parameters. Velocity was determined by computing the resultant speed derived from markers placed bilaterally on the anterior superior iliac spine (ASIS) and posterior superior iliac spine (PSIS) within the pelvis. More specific details of the marker’s paste position are illustrated in Figure 2.

The marker trajectories and ground reaction force data were visually examined for quality, and any gaps in the data were filled using pattern fill functionality in an 8-camera Vicon motion capture system. Subsequently, the raw data were saved as C3D files for further processing utilizing a customized Matlab script. This processing involved generating “trc” and “mot” files, wherein the marker trajectories were filtered with a zero-phase fourth-order Butterworth low-pass filter set at a frequency of 6 Hz. The force data were filtered at 30 Hz [5,10,23]. Initially, the generic model underwent scaling procedures to align with the anthropometric dimensions of each participant, incorporating adjustments for anatomically relevant inertia and moment arms. Subsequently, inverse kinematics techniques were applied to compute the hip, knee, and ankle joint angles.

### 2.4. Statistical Analysis

This study aims to explore the biomechanical characteristics of badminton players during the propulsion step with and without a split-step, as well as the impact of these characteristics on performance efficiency and injury risk. It analyzes the lower limb kinematic characteristics and ground reaction force features during the landing cushioning phase and propulsion phase of the forehand and backhand propulsion steps with and without a split-step, based on the division of badminton textbook movement structures and the relevant literature in sports biomechanics. The landing cushioning and propulsion phases are delineated based on the ground reaction force data from a three-dimensional force plate, with the analysis focusing on the third trial out of five conducted.

Lower limb joint angle and range of motion, kinematic characteristics of the lower limbs at the moment of touchdown and during the contact phase, peak vertical GRF during the contact phase, first vertical peak loading rate, and difference in time to peak vertical reaction force during support phase were chosen for statistical analyses based on the previous literature linked to impact injuries and quality in badminton lunges [12,13,22,24,25].

The foot contact time was defined as the duration from the initial contact to the final take-off of the lunging leg, as determined by the force plate [22,26]. The contact phase of the lunge step was delineated as the duration from the initial heel contact of the landing foot to toe-off, as ascertained through the force plate measurements. Specifically, heel contact and toe-off instances were identified as the moments when the vertical ground reaction force (VGRF) initially exceeded 10 N (heel contact) and subsequently reduced to 10 N (toe-off) [12].

**Figure 3:**
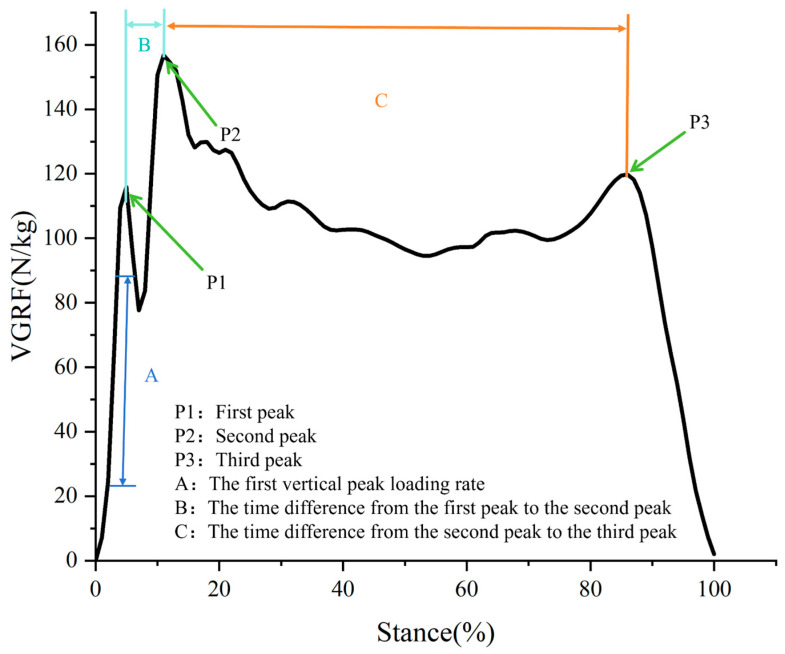
Illustrating the definition of vertical ground reaction force indicators.

The first vertical peak loading rate refers to the steepest slope observed on the vertical ground reaction force (VGRF) curve between consecutive data points from 20% to 80% before the initial peak impact [26,27,28]. The time difference in peak vertical reaction forces during the contact phase refers to the interval between observing three successive peaks in vertical reaction forces: from the first peak to the second peak and from the second peak to the third peak. These peaks correspond respectively to the instances of initial ground contact, support, and take-off phases in vertical reaction forces [22]. The definition of vertical ground reaction force is illustrated in Figure 3.

Owing to their one-dimensional nature, the waveform data of joint angles and GRF were initially interpolated using a cubic spline, resulting in 101 data points representing the entirety of the stance phase (100%) [10]. Before statistical analysis, the normality of variables in this study was assessed using a Shapiro–Wilk test. Additionally, procedures were implemented to control the false discovery rate, particularly for the kinematic data of lower extremity joints. Due to the one-dimensional (1D) nature of joint kinematic trajectories [29,30], the Statistical Parametric Mapping 1D (SPM1D) was applied for the kinematics waveform data analysis of hip, knee, and ankle in three planes and vertical ground reaction force (VGRF) [11]. A paired-sample test was employed to compare the kinematic and ground reaction force data between the lunge steps with and without a split-step for both forehand and backhand movements in archery. All statistical analyses were performed with ORIGIN2022 (OriginLab Corporation, Northampton, MA, USA) and MATLAB R2016a with significance level settings at *p* < 0.05.

## 3. Results

### 3.1. Lower Limb Joint Angle and Range of Motion

Table 1 shows the angles of the hip, knee, and ankle at right foot contact during lunging with and without the split-step. In the FH lunge, the hip abduction and rotation angles in lunging with the split-step are significantly greater than in lunging without the split-step (*p* < 0.05). During the FH lunge, the knee flexion angle at foot contact in lunging with the split-step is significantly less than in lunging without the split-step (*p* < 0.05). In the BH lunge, the knee rotation angle at foot contact in lunging with the split-step is significantly greater than in lunging without the split-step (*p* < 0.05). In the BH lunge, the ankle rotation angle at foot contact in lunging with the split-step is significantly less than in lunging without the split-step (*p* < 0.05).

**Figure 4:**
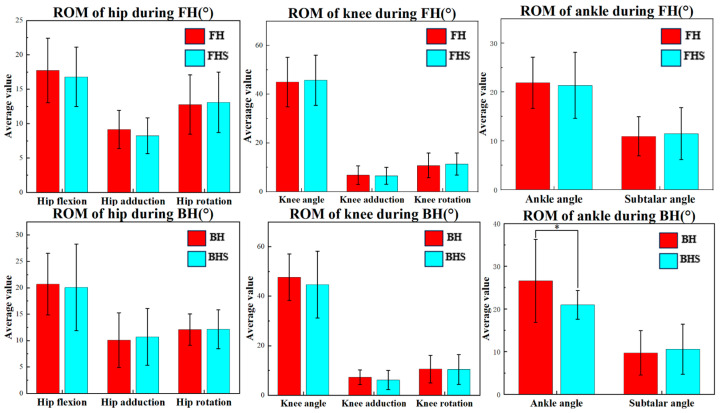
The mean and standard deviation of the range of motion (ROM) of the hip, knee, and ankle angles during the right foot support phase of the FH and BH lunges with and without the split-step. Notes: * indicates significant difference (*p* < 0.05); FH represents forehand lunge without the split-step; FHS represents forehand lunge with the split-step; BH represents backhand lunge without the split-step; BHS represents backhand lunge with the split-step.

Figure 4 depicts the range of motion (ROM) of the hip, knee, and ankle angles during the right foot support phase of lunging with and without the split-step for badminton players. As shown, during the BH lunge, the ankle flexion–extension angle ROM in lunging with the split-step is significantly less than in lunging without the split-step (*p* < 0.05).

**Figure 5:**
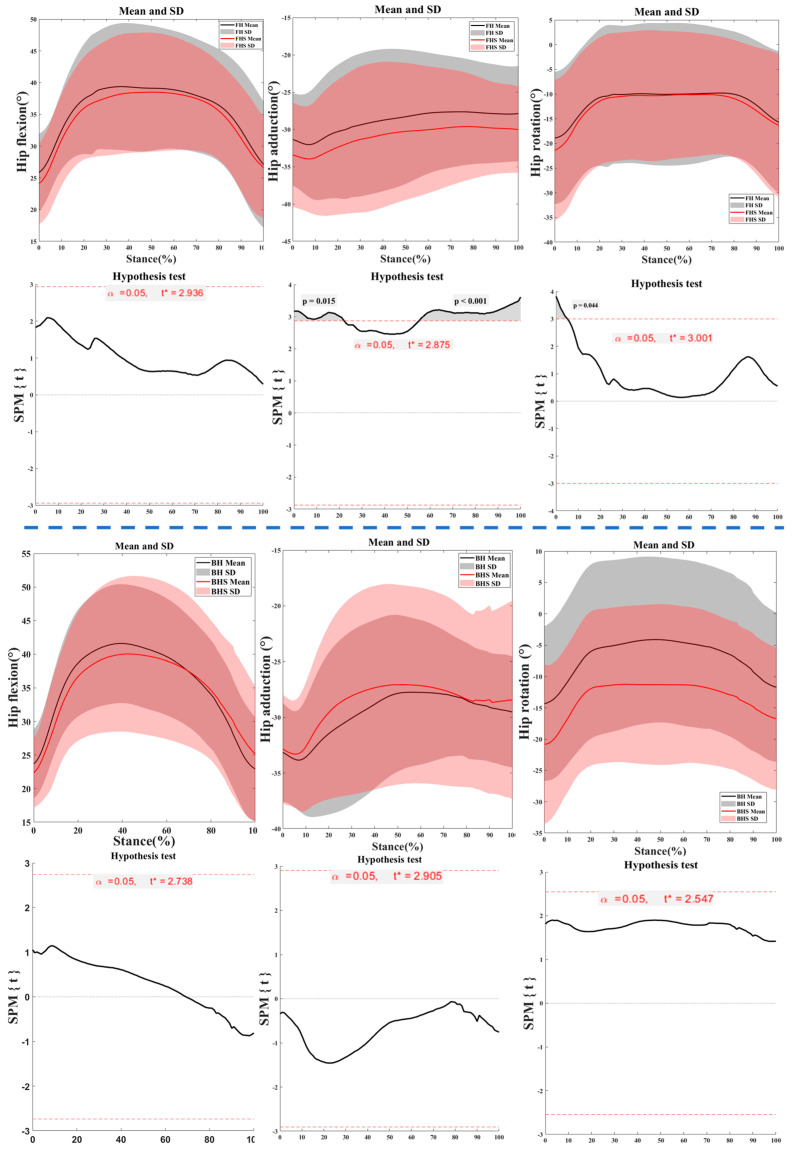
The kinematic characteristics of the hip joint during the right foot support phase of the lunge for FH and BH strides. Notes: FH represents forehand lunge without the split-step; FHS represents forehand lunge with the split-step; BH represents backhand lunge without the split-step; BHS represents backhand lunge with the split-step.

Figure 5 illustrates the kinematic characteristics of the hip joint during the right foot support phase of the lunge for FH and BH strides. There were significant differences observed between the hip flexion angles of the FH lunges with and without the split-step at the 0–22% (*p* = 0.015) and 55–100% (*p* < 0.001) phases. Significant differences were found in the hip rotation angles between the FH lunges with and without the split-step at the 0–5% phase (*p* = 0.044).

**Figure 6:**
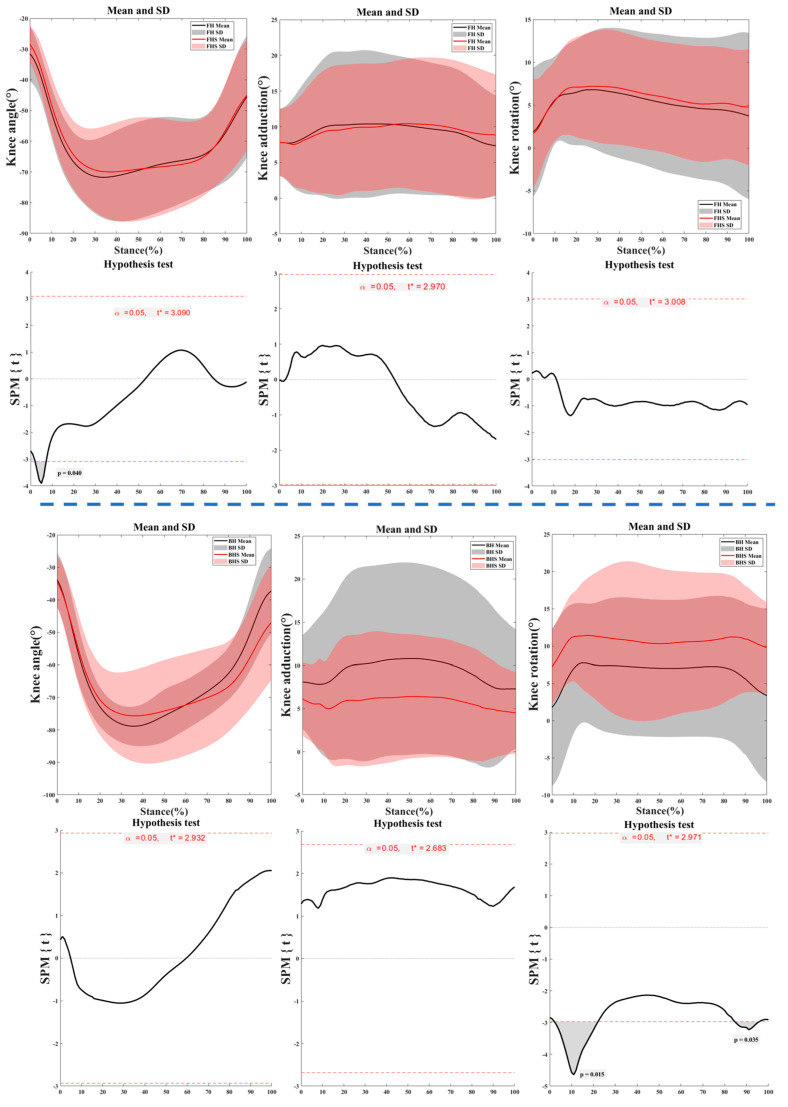
The kinematic characteristics of the knee joint during the right foot support phase of the lunge for FH and BH strides. Notes: FH represents forehand lunge without the split-step; FHS represents forehand lunge with the split-step; BH represents backhand lunge without the split-step; BHS represents backhand lunge with the split-step.

Figure 6 illustrates the kinematic characteristics of the knee joint during the right foot support phase of the lunge for FH and BH strides. The knee joint rotation angles for FH strides with and without the split-step show significant differences at the 2–23% (*p* = 0.015) and 84–95% (*p* = 0.035) phases. For BH strides, the knee joint flexion–extension angles during the right foot support phase of the lunge with and without the split-step exhibit significance at the 2–8% phase (*p* = 0.040).

**Figure 7:**
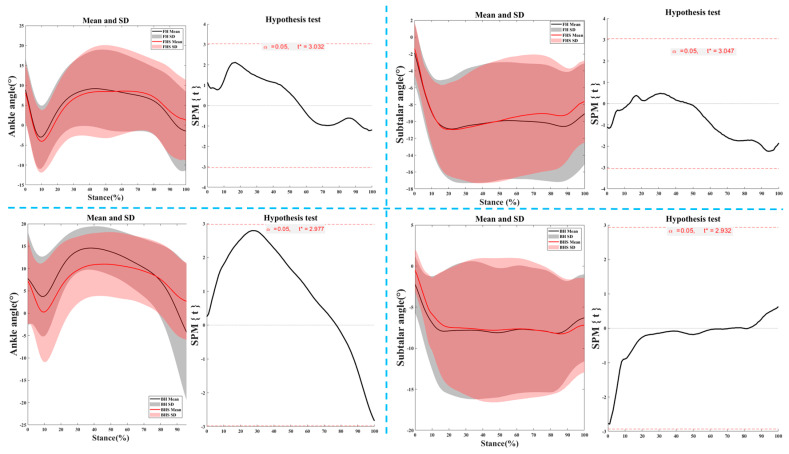
The kinematic characteristics of the ankle joint during the right foot support phase of the lunge for FH and BH strides. Notes: FH represents forehand lunge without the split-step; FHS represents forehand lunge with the split-step; BH represents backhand lunge without the split-step; BHS represents backhand lunge with the split-step.

Figure 7 illustrates the kinematic characteristics of the ankle joint during the right foot support phase of the lunge for FH and BH strides.

### 3.2. Vertical Ground Reaction Force

**Figure 8:**
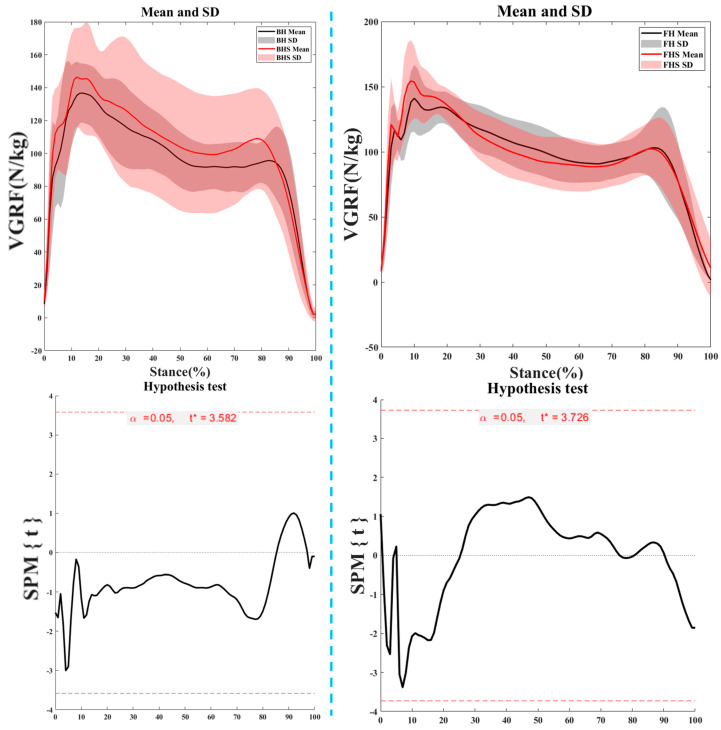
Vertical ground reaction force (VGRF) characteristics during the support phase. Notes: FH represents forehand lunge without the split-step; FHS represents forehand lunge with the split-step; BH represents backhand lunge without the split-step; BHS represents backhand lunge with the split-step.

Figure 8 illustrates the variations in vertical ground reaction force (VGRF) during the support phase of lunges for FH and BH strides. No statistically significant differences were observed.

Table 2 presents the characteristics of the first VGRF peak loading rate during the support phase of the lunge for FH and BH strides. There are differences in the first VGRF peak loading rate between strides with and without the split-step. During the forehand lunge, the first VGRF peak loading rate for strides with the split-step is significantly greater than for strides without the split-step (*p* < 0.05).

Table 3 presents the characteristics of the time difference between the first and second peaks of the vertical ground reaction force (VGRF) during the support phase. For forehand lunges, the time difference between the first and second VGRF peaks was smaller in lunges with the split-step compared to lunges without the split-step, and this difference was statistically significant (*p* < 0.05).

Table 4 presents the characteristics of the time difference between the second and third peaks of the vertical ground reaction force (VGRF) during the support phase. No statistically significant differences were observed.

## 4. Discussion

This study aimed to investigate the biomechanical characteristics of the lower limbs of badminton players during forehand and backhand lunging steps with and without the split-step technique, as well as the impact of these characteristics on movement efficiency and sports injuries. Using a three-dimensional force plate and motion capture system, kinematic and ground reaction force parameters during lunging steps were measured and analyzed for 18 badminton players in both scenarios. The main findings of this study are as follows.

In this study, we observed a significant difference in the hip joint abduction/adduction angle and rotation angle between lunging steps with and without the split-step technique at the moment of right foot contact. This finding underscores the importance of the split-step in the footwork of badminton players [13,31], particularly in the kinematic characteristics of the hip joint. As a crucial pivot point for lower limb movement, variations in hip joint angles directly influence athletes’ stride, speed, and stability. Introducing the split-step may provide athletes with greater stride length and faster movement speed by increasing the range of motion in the hip joint, thus offering an advantage in badminton matches [32]. The increase in hip joint abduction/adduction angle implies that athletes can utilize the hip muscles more effectively during lunging steps, which may be related to the pre-activation performed during the split-step [8]. Pre-activation enhances muscle readiness, allowing greater force production and faster speed during subsequent lunging steps. Additionally, the increase in the hip joint rotation angle may be associated with athletes adjusting their body orientation to adapt to the flight trajectory of the shuttlecock [33]. Rapid adjustments in body orientation are crucial for successful shuttlecock retrieval in badminton, and the flexible movement of the hip joint provides the necessary biomechanical foundation [34].

Regarding the ankle joint, we found that during the landing phase of the backhand lunges with the split-step, the rotation angle of the ankle joint was significantly smaller compared to backhand lunges without the split-step. Additionally, the ankle joint dorsiflexion angle’s range of motion (ROM) was significantly smaller during backhand lunges with the split-step compared to those without the split-step. This suggests that during backhand lunges, the split-step may reduce the mobility of the ankle joint, thereby enhancing ankle joint stability and effectively preventing sports injuries [20,35]. Moreover, the stability and flexibility of the ankle joint are crucial for the coordinated movement of the entire lower limb chain [36,37]. Future research could further investigate the role of the ankle joint in different footwork patterns and explore methods to optimize ankle joint function through training.

Although significant differences were observed in the kinematic characteristics of the hip and ankle joint during the split-step, no significant changes were noted in the kinematic characteristics of the knee at the moment of ground contact. This may suggest that during the initial phase of lunging steps, the motion of the knee is primarily influenced by ground reaction forces and shifts in the body’s center of mass rather than by the execution of the split-step. This finding is consistent with previous studies on the kinematics of lunging steps [10,31], indicating that the knee joints primarily serve a buffering and stabilizing role during stride transitions. In contrast, the hip joint plays a predominant role in dynamic stride adjustments [10].

In this study, we conducted a detailed analysis of badminton players’ lower limb kinematic characteristics during forehand and backhand lunging steps, particularly during the right foot support phase, comparing the differences between lunging steps with and without the split-step technique. The results revealed significant effects of the split-step on athletes’ lower limb kinematic characteristics, particularly at specific stages of hip and knee joint activity. Firstly, the hip joint abduction/adduction angle was significantly greater during forehand lunging steps with the split-step than without, especially during the movement’s early (0–22%) and late (55–100%) phases. This suggests that the split-step may provide athletes with a greater range of motion in the hip joint, thereby aiding in increasing stride length and enhancing movement speed. Such kinematic characteristics of the hip joint are crucial for badminton players to adjust their body posture and prepare for hitting the shuttlecock during rapid movements. This advantage may directly impact their performance and match outcomes, particularly during critical game moments [13,38]. Secondly, the hip joint rotation angle was significantly smaller during forehand lunging steps with the split-step than without, especially during the movement’s early (0–5%) phase. This may indicate that athletes are more inclined to adjust their body orientation through hip joint abduction/adduction movement rather than rotation during the split-step. This strategy may help athletes rapidly adapt to the optimal hitting position while maintaining stability [39].

Regarding the knee joint, the rotation angle during backhand lunging steps with the split-step was significantly greater than without at specific stages (2–23% and 84–95%). This suggests that the split-step may facilitate a larger range of rotation at the knee joint during the lunging step, which is crucial for athletes to maintain balance and adjust stride rhythm during movement [31,40]. The knee joint flexion/extension angle exhibited significant differences during forehand lunging steps, with the split-step at the 2–8% stage. This may reflect that the split-step provides additional propulsion for athletes during the initial push-off phase, resulting in greater torque during knee joint flexion [21,31].

In this study, we analyzed badminton players’ ground reaction force (GRF) characteristics during forehand and backhand lunging steps with and without a split-step. The results revealed the impact of the preparatory step on the time difference between GRF peaks and loading rates, which holds significant implications for understanding the biomechanics of badminton footwork. Firstly, in forehand lunging steps, the time difference between the first and second GRF peaks was significantly shorter in steps with a split-step than those without. This suggests that the preparatory step facilitates a quicker transition from heel contact to full foot contact during the support phase, potentially enhancing the athlete’s ability to efficiently absorb and transmit force, consequently generating greater propulsion during the push-off phase [10,11,41]. This ability to rapidly adjust footwork is crucial for swiftly reaching hitting positions and maintaining balance during badminton matches [5]. However, in forehand lunging steps, no significant difference was observed in the time difference between the second and third GRF peaks with and without a split-step. This suggests that the split-step may have a limited optimization effect on the time difference between GRF peaks during the push-off phase, potentially because the primary goal during this phase is to generate sufficient force to complete the step, and the time difference between GRF peaks may not be the primary determinant of performance during this phase.

Additionally, during forehand lunges, the first vertical GRF peak loading rate of lunges with the split-step was significantly greater than those without the split-step. This suggests that the split-step enhances the loading rate of vertical GRF upon foot contact, potentially aiding in shortening the duration of the lunge motion. This is consistent with findings from previous studies. Previous research has shown that the split-step can improve initial acceleration and stride efficiency in tennis players and reaction speed in soccer goalkeepers during penalty kicks [16,42].

When discussing the limitations of this study, it is essential to acknowledge that the sample size may affect the generalizability and reliability of the results. Due to the small sample size, our findings may only partially represent some biomechanical characteristics of lower limb movements during lunging steps in all badminton players. Additionally, individual differences among athletes, including skill level, training background, physical condition, and age, could significantly influence GRF characteristics, and these factors needed to be adequately considered in this study. Therefore, future research should aim to increase the sample size and account for individual differences among athletes to understand better the effects of preparatory steps on the biomechanical characteristics of lunging steps.

Furthermore, this study only focused on forehand and backhand lunging, while badminton players execute various steps during matches. To comprehensively understand the effects of split-steps, future research should include more types of steps, such as lateral steps and jumping steps, as well as different step executions in various match situations, such as fast counterattacks, defensive transitions, etc.

Moreover, this study primarily focused on the time difference between GRF peaks and loading rates without thoroughly analyzing the dynamic changes of GRF throughout the entire step cycle. Future research could investigate the effects of preparatory steps on the distribution and transmission of forces throughout the entire step cycle through finer temporal resolution and more comprehensive GRF analysis.

## 5. Conclusions

This study analyzed the biomechanical effects of incorporating the split-step in both forehand (FH) and backhand (BH) lunging steps in badminton. The results indicated that the split-step significantly improves the efficiency and velocity of the FH lunging step. This improvement is characterized by increased hip joint angles and decreased knee flexion angles at the moment of foot contact. Moreover, the study found an enhanced loading rate of the initial GRF peak and a reduced time interval between the first and second GRF peaks during the FH lunge with the split-step, further supporting the beneficial role of the split-step in enhancing stride efficiency. However, the impact of the split-step in the BH lunging step was not as pronounced, which may point to the influence of other factors on stride efficiency and stability. In summary, the split-step plays a vital role in optimizing performance for FH lunging actions. In contrast, the effectiveness of the split-step in BH actions warrants further exploration and refinement in training approaches. These insights offer a scientific foundation for athletes and coaches to improve technical training and movement efficiency in badminton.

## Figures and Tables

**Table 1:** The mean, standard deviation, and 95% confidence interval of the hip, knee, and ankle angles at the moment of right foot contact during FH and BH lunges with and without the split-step.

Joint	Variables	FH	FHS	95%CI	*p*	BH	BHS	95%CI	*p*
Hip	Flexion/Extension	25.85 ± 6.21	24.10 ± 6.48	[−0.21, 3.70]	0.08	23.68 ± 5.25	22.33 ± 5.28	[−1.31, 4.01]	0.30
	Abduction/Adduction	−31.34 ± 6.32	−33.40 ± 7.11	[0.73, 3.39]	0.004 *	−33.14 ± 4.54	−32.82 ± 4.98	[−2.27, 1.63]	0.74
	Internal/External rotation	−18.93 ± 13.64	−21.43 ± 14.50	[1.16, 3.84]	0.001 *	−14.32 ± 12.61	−20.80 ± 13.00	[−0.93, 13.89]	0.08
Knee	Flexion/Extension	−31.54 ± 9.11	−28.48 ± 6.16	[−5.39, −0.73]	0.01 *	−33.84 ± 8.44	−34.39 ± 8.09	[−2.07, 3.16]	0.06
	Abduction/Adduction	7.81 ± 4.73	7.82 ± 4.83	[−0.66, 0.65]	0.99	8.08 ± 5.55	6.15 ± 4.31	[−1.18, 5.04]	0.21
	Internal/External rotation	1.86 ± 7.67	1.68 ± 6.52	[−1.43, 1.80]	0.82	1.76 ± 1.79	1.20 ± 5.05	[−9.41, −1.47]	0.01 *
Ankle	Flexion/Extension	8.87 ± 8.05	8.09 ± 7.42	[−0.63, 2.20]	0.26	7.76 ± 10.68	7.19 ± 9.80	[−4.14, 5.29]	0.80
	Internal/External rotation	−1.74 ± 3.45	−1.36 ± 3.18	[−1.28, 0.51]	0.39	−2.25 ± 3.54	−0.52 ± 2.63	[−3.01, −0.43]	0.01 *

Note: * indicates significant difference (*p* < 0.05); FH represents forehand lunge without the split-step; FHS represents forehand lunge with the split-step; BH represents backhand lunge without the split-step; BHS represents backhand lunge with the split-step.

**Table 2:** The characteristics of the first VGRF peak loading rate during the support phase (unit: N/kg%).

Footwork	Mean ± SD	95%CI	*p*
FH	33.30 ± 13.40	[−13.67, 0.13]	0.04 *
FHS	40.06 ± 15.91		
BH	29.96 ± 15.23	[−5.37, 11.04]	0.48
BHS	29.24 ± 12.25		

Note: * indicates significant difference (*p* < 0.05); FH represents forehand lunge without the split-step; FHS represents forehand lunge with the split-step; BH represents backhand lunge without the split-step; BHS represents backhand lunge with the split-step.

**Table 3:** Time difference between the first and second VGRF peaks during the support phase (unit: %).

Footwork	Mean ± SD	95%CI	*p*
FH	9.9 ± 4.24	[0.21, 4.59]	0.03 *
FHS	7.5 ± 2.78		
BH	7.66 ± 2.38	[−2.29, 0.51]	0.12
BHS	8.55 ± 3.05		

Notes: * indicates significant difference (*p* < 0.05); FH represents forehand lunge without the split-step; FHS represents forehand lunge with the split-step; BH represents backhand lunge without the split-step; BHS represents backhand lunge with the split-step.

**Table 4:** Time difference between the second and third GRF peaks during the support phase (unit: %).

Footwork	Mean ± SD	95%CI	*p*
FH	67.35 ± 10.79	[−9.95, 0.25]	0.06
FHS	72.2 ± 8.33		
BH	70.77 ± 7.97	[−2.66, 7.10]	0.35
BHS	68.56 ± 6.58		

Notes: FH represents forehand lunge without the split-step; FHS represents forehand lunge with the split-step; BH represents backhand lunge without the split-step; BHS represents backhand lunge with the split-step.

## Data Availability

The data related to the results and findings of this study will be available upon reasonable request to the corresponding author.
